# Symmetric instability drives exchange between surface and bottom waters in a coastal front

**DOI:** 10.1126/sciadv.aeb9841

**Published:** 2026-05-08

**Authors:** Mareike Körner, Jesse M. Cusack, Jonathan Nash, R. Kipp Shearman, Leif N. Thomas, Jennifer MacKinnon, Fucent Hsuan Wei Hsu, John Taylor, Jinliang Liu, James P. Hilditch

**Affiliations:** ^1^ College of Earth, Ocean, and Atmospheric Science, Oregon State University, Corvallis, OR, USA.; ^2^ Department of Earth System Science, Stanford University, Stanford, CA, USA.; ^3^ Scripps Institution of Oceanography, University of California, San Diego, La Jolla, CA, USA.; ^4^ Department of Applied Mathematics and Theoretical Physics, University of Cambridge, Cambridge, UK.

## Abstract

The coastal ocean in the northern Gulf of Mexico is highly productive and socioeconomically important. Here, stratification can inhibit vertical exchange, with consequences for ecosystem health and hypoxia. Previous work emphasized the role of wind-driven turbulent mixing as a mechanism to overcome the stratification barrier. We identify symmetric instability (SI) as an additional and more energy-efficient pathway linking surface and bottom waters. In high-resolution observations, diagonal bands of overturning motions, a telltale sign of SI, connect the sea surface to the bottom and produce intrusions of temperature and oxygen anomalies. Overturning persists for 2 days after instability-favorable wind ceases. During this time, vertical advective fluxes exceed turbulent fluxes by an order of magnitude, ventilating low-oxygen bottom waters and transporting surface heat downward. Our results show that SI facilitates vertical exchange that can outlive direct wind forcing, highlighting an instability-driven mechanism that may be important in coastal oceans more generally.

## Teaser

Observations reveal the spatial structure of symmetric instability and its role in vertical exchange of oxygen and temperature.

## INTRODUCTION

Nearly half of the global population lives in coastal regions (*1*), depending on the ocean for food, livelihoods, and climate resilience (*2*, *3*). In the northern Gulf of Mexico, the ecosystem faces multiple stressors, including rising sea levels and elevated ocean heat content, which intensify hurricanes and exacerbate storm surge and flooding risks (*4*–*6*). The region also experiences one of the world’s largest seasonal hypoxic zones (*7*–*9*), which threatens benthic communities and the local shrimp fisheries (*10*). It is driven by inputs from the Mississippi-Atchafalaya River, which delivers fresh water and nutrients, fueling high productivity and generating strong vertical and horizontal density gradients that stratify the coastal ocean (*8*). These conditions can lead to oxygen depletion when persistent stratification causes respiratory oxygen consumption to outpace ventilation (*7*–*9*, *11*). In the northern Gulf of Mexico, hypoxia typically develops in late spring as stratification strengthens due to increased river discharge and surface warming, reaches its maximum extent in summer, and is eroded in fall by ventilation driven by enhanced wind-driven mixing and cooling (*7*, *12*, *13*).

Previous works have emphasized wind-driven turbulent mixing, for example, during storms, as a primary mechanism terminating hypoxic conditions in the northern Gulf of Mexico (*9*, *14*). Such events can erode stratification and ventilate bottom waters. Note that tidal forcing in the northern Gulf of Mexico is relatively weak (*15*, *16*) and thus are not thought to provide a major source of vertical mixing or ventilation in this region. However, other physical mechanisms, like submesoscale processes, can drive vertical exchange, even when winds are too weak to break down the stratification barrier. Elucidating these vertical exchange mechanisms is critical for understanding coastal systems and their resilience to future change, not only in relation to hypoxia but also for heat content, nutrient supply, and primary productivity. In this study, we show that symmetric instability (SI), a type of submesoscale instability, provides an additional pathway for vertical exchange along a freshwater front in the northern Gulf of Mexico.

Submesoscale processes, characterized by horizontal scales of 0.1 to 10 km and timescales of hours to days, occupy a dynamical regime where both stratification and the Earth’s rotation matter, but neither dominate (*17*–*19*). SI is a specific submesoscale process that develops when there is an imbalance between gravitational and Coriolis forces (*20*), typically at fronts with strong vertical shear and horizontal density gradients (*21*, *22*). It is characterized by small-scale, slantwise overturning motions along isopycnal surfaces (*23*). SI extracts energy from the mean geostrophic flow, dissipates energy through secondary shear instabilities, and can thus lead to elevated turbulence levels (*24*, *25*). Although SI has been studied extensively in theory and numerical models (*20*, *22*, *25*–*29*), observational evidence is largely indirect (*21*, *24*, *30*–*33*) because their small spatial and temporal scales make it difficult to capture in the field. Most observational studies identify the necessary conditions for SI and find associated elevated dissipation rates but do not resolve the overturning cells or their advective impact. Consequently, their net contribution to vertical exchange and its broader role in shaping marine ecosystems remain uncertain.

To investigate how submesoscale dynamics interact with wind-driven internal waves in stratified coastal environments, intensive observations were acquired in the northern Gulf of Mexico during the National Science Foundation (NSF) Submesoscale Under Near-Resonant Inertial Shear Experiment (SUNRISE). Data from this campaign reveal SI along a gently sloping surface-to-bottom freshwater front. We characterize the dynamics and structure of this instability event, identify the conditions under which SI cells emerge and persist, and demonstrate how top-to-bottom overturning redistributes heat and oxygen within highly stratified coastal waters.

## RESULTS

### SI at the front

In summer 2021, a series of coordinated two-ship surveys captured the three-dimensional evolution of frontal features. For most of the measurement period, both ships conducted parallel cross-front sections spaced ~1 km apart. On both ships, velocity was measured with high-frequency acoustic Doppler current profilers (ADCPs), providing fine vertical resolution. Hydrography, turbulence, and dissolved oxygen were measured by rapidly sampling with free-falling profilers from the stern of each ship, resulting in profiles approximately every 100 m. This sampling approach enabled synoptic observations of horizontal and vertical gradients and provides a detailed view of frontal processes with high spatial and temporal resolution (see Materials and Methods for more details).

**Fig. 1.**
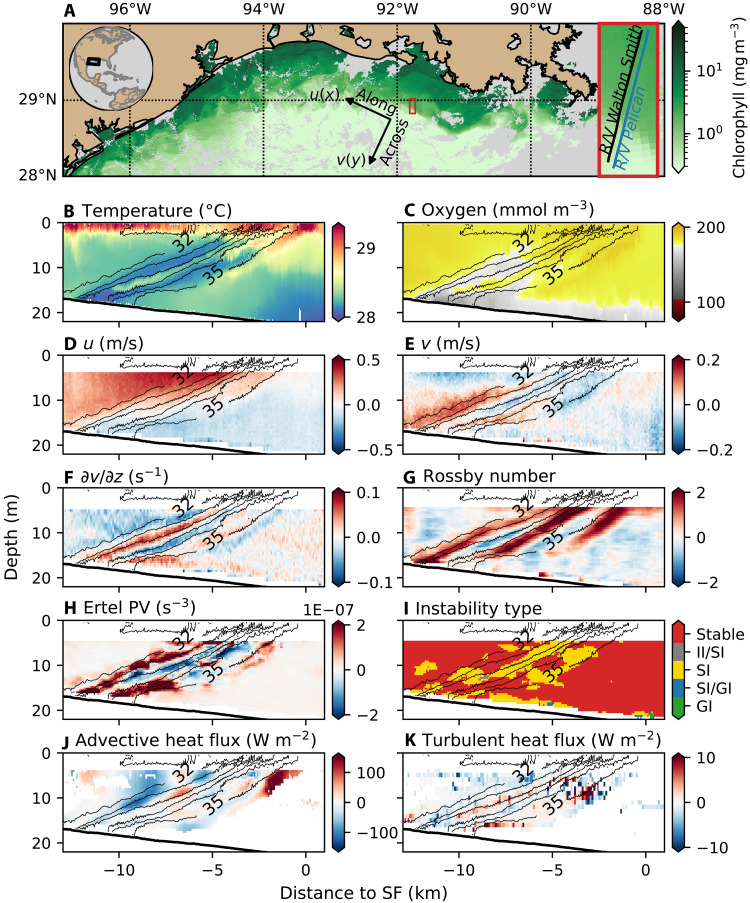
SI at the freshwater front. (**A**) Satellite-derived chlorophyll a concentrations in the northern Gulf of Mexico on 1 July 2021. The red box shows the cross-front survey, where the *R/V Pelican* and *R/V Walton Smith* conducted parallel transects. Arrows indicate the orientation of the across-front and along-front directions along with the definition of the coordinate system. (**B** to **K**) Cross-front sections are plotted as a function of depth and distance to the surface front (SF). Panels show the (B) temperature, (C) oxygen, (D) along-front velocity (*u*), (E) across-front velocity (*v*), (F) vertical shear of the across-front velocity (∂v/∂z), (G) relative vorticity normalized by the Coriolis parameter (*f*), (H) Ertel PV, and (I) Instability classification (see Supplementary Text), with colors indicating stable flow (red), inertial instability (II)/SI (gray), SI (yellow), SI/gravitational instability (GI) (blue), and gravitational instability (green). (J) shows the vertical advective heat flux, and (K) shows the vertical turbulent heat flux. Thin black lines in (B) to (K) show salinity contours every 0.6 practical salinity unit (PSU); the 32 and 35 PSU isohalines are labeled.

One of the transects crossing the front is shown in Fig. 1. Salinity changes by 3 g kg^−1^ across the ~2-km-wide front and is the dominant control on water density (contours in Fig. 1, B to K, and fig. S1). The front is characterized by a 0.5 m s^−1^ along-front current (Fig. 1D), with small-scale banded variability superimposed. Temperature and oxygen sections reveal alternating bands of warm, oxygen-rich and cooler, oxygen-poor water aligned along sloped density surfaces (Fig. 1, B and C), suggestive of layered intrusions. The across-front velocity field (Fig. 1E) exhibits a series of flow reversals in the region of the front, which are more clearly visible in its vertical gradient (Fig. 1F). The alignment of the temperature, oxygen, and velocity signals indicates the presence of small-scale overturning cells spanning the entire water column.

The slantwise banded structures resemble overturning cells interpreted as SI in numerical simulations (*23*, *34*). To assess whether our observed signals are consistent with SI, we diagnose the flow stability using the hydrostatic Ertel potential vorticity (PV), $q=\left(f\hat{k}+\nabla \times u\right)\cdot \nabla b$, where *f* is the Coriolis parameter, $\hat{k}$ is the vertical unit vector, u=(u,v,0) is the velocity, and $b=-\mathrm{g\rho}/\rho_{0}$ is the buoyancy (*g* is the acceleration due to gravity and ρ is the density). The parallel ship sections provide a three-dimensional view of the flow field, allowing us to compute all dominant terms of PV using a two-ship solution (see Materials and Methods). We find that there are large patches of negative PV in the frontal zone (Fig. 1H), indicating dynamic instability that is a necessary condition for SI (*20*, *23*). To further distinguish among possible instability types, we apply a classification method based on the individual PV terms (see Supplementary Text for details), which confirms that SI is the prevailing mode of instability expected to be active at the front (Fig. 1I). In addition, the observed banded structures align with isopycnals (Fig. 1F), consistent with linear theory predictions that the fastest-growing mode of SI has streamlines parallel to isopycnals (*20*, *21*, *23*). The expected wavelength of the SI cells is $L_{\text{SI}}=H/s_{\rho }$, where *H* is the depth of the SI layer and *s*_ρ_ the slopes of the isopycnals (*27*, *30*). The isopycnal slopes of the section presented in Fig. 1 is ~2.5 × 10^−3^, and the SI layer depth is 10 m (depth of the negative PV layer). Thus, the expected wavelength *L*_SI_ is 4 km similar to the size of the observed SI cells (Fig. 1).

Vertical exchange by SI can occur through advection by the larger-scale overturning circulation of the primary instability and turbulent mixing generated by smaller-scale secondary instabilities. Previous studies have primarily emphasized the turbulent pathway when assessing the potential impacts of SI on ecosystems (*22*, *24*, *35*). Here, we show that the advective pathway can also play a dominant role in redistributing tracers. In our observations, banded structures in temperature and oxygen fields are aligned with sloping density surfaces at the front, span the whole water column, and exhibit strong cross-band gradients (Fig. 1, B and C), suggesting that, in this case, advection is the dominant mechanism of top-to-bottom transport. Quantitative estimates of the vertical heat flux support this interpretation: Advective heat fluxes reach −150 W m^−2^, more than an order of magnitude greater than turbulent fluxes, which are around −10 W m^−2^ (Fig. 1, J and K) (see Materials and Methods for details on the flux calculations). Note that turbulent heat fluxes exhibit both positive and negative values, reflecting sign changes in the vertical temperature gradient associated with layered intrusions. Gaps in the flux estimates arise because we restrict the analysis to the stratified frontal region to isolate SI-driven exchange.

### The forcing mechanism and life cycle

We now examine the boundary forcing mechanisms driving SI at the freshwater front. In general, winds that blow in the direction of the geostrophic current, so-called “down-front winds,” can trigger SI by inducing a cross-front Ekman transport. Similarly, friction at sloping bathymetry can also cause a bottom boundary layer (BBL) Ekman transport. In both cases, the Ekman transport may drive convective instability that steepens isopycnal slopes and reduces PV (*24*, *27*, *28*, *34*, *36*–*38*). The Ekman buoyancy flux (EBF) quantifies the magnitude of the buoyancy change caused by Ekman transport ($\text{EBF}=M_{E}\cdot \nabla_{h}b$ with Ekman transport $M_{E}$ and horizontal buoyancy gradient $\nabla_{h}b$). Although the EBF is not a buoyancy flux in the traditional sense (e.g., heat or freshwater exchange), it quantifies the role of Ekman transport in modifying buoyancy at the boundaries. Thus, the EBF acts like a flux capable of reducing PV and triggering SI (*27*).

**Fig. 2.**
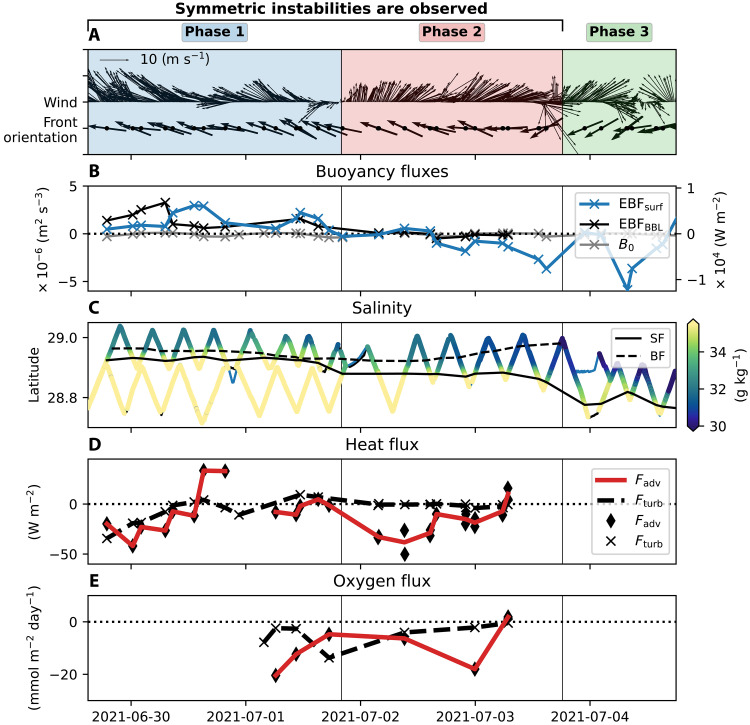
Time evolution of atmospheric forcing, frontal properties, and vertical fluxes. (**A**) Time series of wind vectors and front orientation vectors. Colors indicate different observational phases. (**B**) EBFs from the surface (blue) and BBL (black) averaged for each section between 0 and −10 km distance to the surface front. Gray line shows the net surface buoyancy fluxes averaged for the respective time period of the section measurements. Buoyancy fluxes are plotted in both units of m^2^ s^−3^ and W m^−2^. (**C**) Salinity (color) averaged over the top 2 m along ship tracks, with the surface front position (solid black) and bottom front (dashed black). (**D**) Heat fluxes due to advection (red line, diamonds) and turbulent mixing (black dashed line, crosses). (**E**) Oxygen fluxes due to advection (red line, diamonds) and turbulence (black dashed line, crosses). Diamonds and crosses represent individual flux estimates for each section and ship. The red solid and black dashed lines connect these estimates; when both ships were present, it represents the mean of their estimates. Shaded background colors in (A), and vertical lines in (A) to (E) indicate the three observational phases.

We examine the wind forcing between 29 June and 3 July (Fig. 2A) to show that SI occurs under a range of wind regimes and EBFs. During this 5-day period, all 25 transects exhibit negative PV consistent with SI (figs. S3 and S4). The cross-front section presented in Fig. 1 is not an isolated case.

**Fig. 3.**
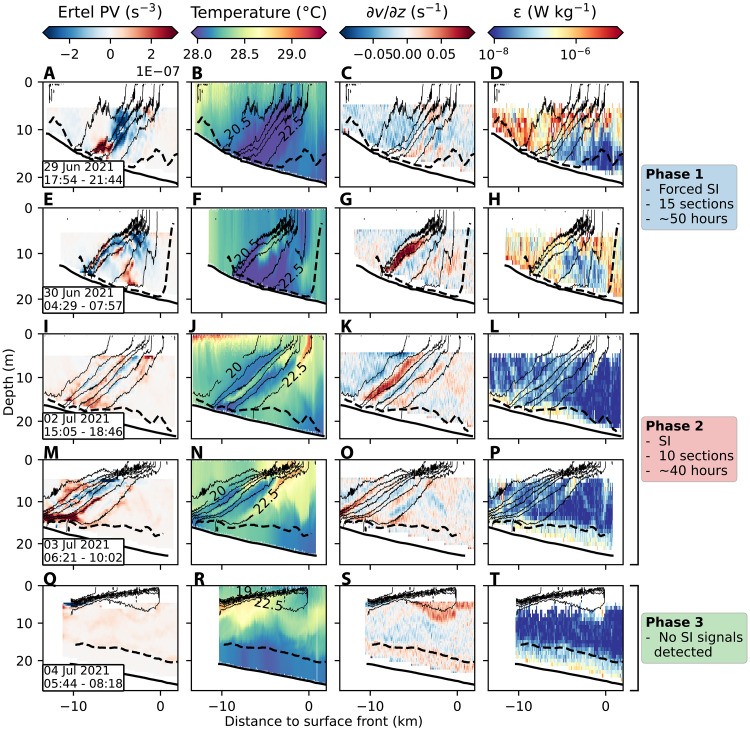
Cross-front sections during the different phases. (**A** to **T**) From left to right, each column shows the Ertel PV, temperature, vertical shear of across-front velocity (∂v/∂z), and turbulent kinetic energy dissipation rate (ϵ). Each row represents a distinct section, ordered chronologically from top to bottom. The time [universal time coordinated (UTC)] of each measured section is given in the lower left corner in the left column. Thin black lines show potential density contours every 0.5 kg m^−3^; selected isopycnals are labeled in the temperature plots. The dashed lines denote the BBL. Brackets on the right indicate the observational phases associated with the phases introduced in Fig. 2. Cross-front sections presented here display examples of sections measured during the respective phases; for more sections, refer to figs. S1 to S4.

The evolution of the front and instabilities can be separated into three phases characterized by different wind forcing (Fig. 2A). During phase 1, strong down-front winds drive surface water toward the coast, pushing the front shoreward and steepening it (Fig. 2C). These winds cause the positive EBF (destabilizing), which peaks on 30 June at ~3 × 10^−6^ m^2^ s^−3^, comparable in magnitude to the convective turbulence produced by a surface heat flux of ~6000 W m^−2^ (Fig. 2B). On 1 July, winds weaken and shift direction, marking the onset of phase 2. During this phase, the EBF decreases and becomes negative (stabilizing). Despite stabilizing atmospheric forcing, SI signatures persist for an additional ~40 hours (Fig. 3 and figs. S3 and S4). In phase 3, winds become variable and change direction, displacing the front offshore (Fig. 2C). SI signatures are no longer detected during phase 3 (Fig. 3 and figs. S3 and S4).

Because SI overturning cells span the full water column, we also consider the influence of the BBL forcing. Figure 2B shows that, during phase 1, the bottom EBF is comparable in magnitude to the surface EBF, indicating that both contribute to front destabilization and the generation of SI. As surface forcing weakens and becomes stabilizing in phase 2, the bottom EBF weakens.

In addition to boundary stresses, surface buoyancy fluxes can also modulate flow stability influencing PV. However, we find that net surface buoyancy fluxes (heat and freshwater exchange) remain small throughout all phases, with only minor destabilizing effects during nighttime cooling (order of 10^−7^ m^2^ s^−3^; Fig. 2B).

Together, these results demonstrate that SI is active across a range of atmospheric forcing. We identify two distinct regimes: a forced regime during phase 1, when atmospheric and bottom-driven forcing both actively destabilize the front, and a free-evolving or weakly forced regime during phase 2, when surface forcing ceases but the instability signals persists. A natural question spurred by these results is: Why does SI persist when the destabilizing forcing ends?

**Fig. 4.**
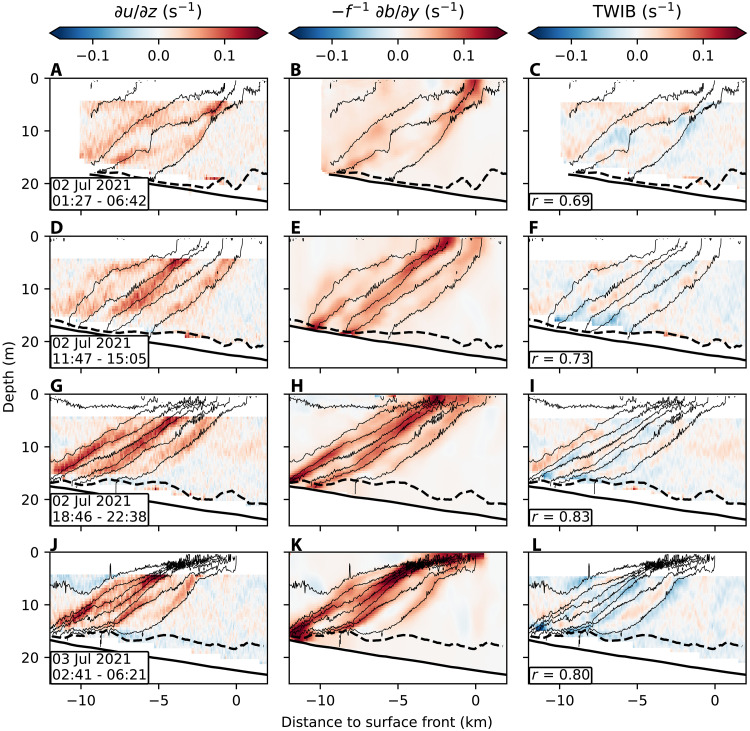
Cross-front sections of components of the thermal wind balance and its residual. (**A** to **L**) The left column shows the observed vertical shear of the along-front velocity (∂u/∂z), the middle column shows the buoyancy term of the thermal wind balance ($-f^{-1}\text{∂}b/\text{∂}y$), and the right column shows the TWIB, which is defined as the difference between the observed shear and the buoyancy term ($\text{TWIB}=\text{∂}u/\text{∂}z-f^{-1}\text{∂}b/\text{∂}y$). Each row corresponds to a distinct cross-front section, with the date and time (UTC) range noted in the left panel. Thin black lines represent isopycnals, and dashed black lines denote the BBL depth. Residual panels include the Pearson correlation coefficient (*r*) between thermal wind balance estimates and observed shear. Agreement between observed shear and buoyancy forcing indicates when the front is in approximate thermal wind balance.

The circulation cells that characterize SI persist for nearly 40 hours after the wind shifts from destabilizing to weak or stabilizing conditions (phase 2). The front remains close to a thermal wind balance throughout this period, including the along-front flow associated with the SI motions (Fig. 4). The diagnosed thermal wind imbalance (TWIB) is weak and vertically distributed throughout the water column (Fig. 4, C, F, I, and L), which is consistent with a slowly evolving SI circulation. The end of phase 2 is marked by the onset of stronger and more variable wind (Fig. 2A). Note that, at the end of phase 2, the flow still exhibits the conditions required for SI to persist. PV remains negative and overturning cells are still evident, yet signals of overturning signals vanishes. This apparent shutdown of SI signatures may reflect the influence of external forcing, most likely the onset of stronger and more variable upwelling-favorable winds that disrupt the instability-driven circulation. Alternatively, the disappearance of SI signals may be related to spatial sampling. The ship moves offshore to follow the surface expression of the front (Fig. 2B), and as the frontal slope weakens, the measurements no longer sample the full frontal structure (Fig. 3, Q to T). Consequently, SI may remain active outside the sampled region.

The theoretical timescale for SI to erode the thermal wind shear depends on the strength of the stratification and the front (*29*). In strongly stratified fronts, SI overturning motions tend to align with gradually sloping isopycnals. This geometry limits vertical motion and momentum fluxes, making the instability less effective at mixing away the frontal shear. The observed front is characterized by strong stratification as the inverse isopycnal slope $N^{2}/M^{2}$, where $N^{2}=\text{∂}b/\text{∂}z$ is the vertical buoyancy gradient and $M^{2}=|\nabla_{h}b|$ is the horizontal buoyancy gradient, is of order 1000. The frontal strength $\Gamma =M^{2}/f^{2}$ is of order 1000. Although these parameter values exceed those considered by Wienkers *et al.* (*29*), we can use their scaling to estimate the decay time of the observed SI signals. We estimate the SI mixing timescale for each cross-front section individually (fig. S5). Although there is variability between individual sections, the distribution indicates long-lived SI motions, with a mean decay timescale of 5.8 days, underlining the persistence of SI-driven circulations in this strongly stratified front.

Alternatively, SI may have transitioned into a so-called fossil mode, a slowly evolving remnant that can persist even after the main instability subsides (*25*). This fossil mode may account for the continued SI signatures despite the balanced appearance of the flow.

Overall, our observations demonstrate that SI can remain dynamically important well beyond the forcing period, with important implications for material transport. The observed persistence of SI, together with the maintenance of thermal wind balance, highlights an important and intriguing aspect of its dynamics that warrants further studies.

### Vertical fluxes and their drivers

To assess the role of SI in vertical exchange, we quantify vertical heat and oxygen fluxes, distinguishing between their turbulent and advective contributions. Turbulent fluxes are calculated from microstructure measurements (*39*) and represent vertical mixing driven by small-scale turbulence. Vertical advective fluxes reflect the transport of temperature and oxygen anomalies by the overturning circulation associated with SI. Flux estimates are restricted to sections in which the full frontal structure, spanning from the surface to the bottom, is sampled. Strong net fluxes occur after the instability-favorable wind forcing ceases (phase 2) and are predominantly caused by advection (Fig. 2, D and E). In contrast, phase 1 features more variable fluxes, with comparable contributions from turbulence and advection.

During the forced SI phase (phase 1), winds push the front shoreward, creating steep isopycnals and top-to-bottom mixing (Fig. 3, A to H). Although regions of negative PV indicative of SI are present throughout, the overturning cells do not always exhibit strong temperature anomalies (Fig. 3B). This reflects the effects of intense mixing, which is capable of eroding signals quickly. Vertical turbulent heat fluxes peak on 29 June at −28 W m^−2^ (equivalent to −0.6 K day^−1^) and decline afterward. Vertical advective heat fluxes during phase 1 show substantial variability across sections, ranging from −40 to 33 W m^−2^ (Fig. 2D). Strong along-front velocities and high variability between successive sections suggest that a simple two-dimensional interpretation may not apply during phase 1.

During the transition to upwelling-favorable winds (phase 2), signals of SI become notably persistent. All 10 cross-front sections recorded during this phase exhibit clear signatures of SI, including coherent bands in the vertical shear of the cross-front velocity and temperature fields (Fig. 3 and figs. S2 to S4). Note that the section shown in Fig. 1 is also part of phase 2. The consistency across sections suggests that along-front variability is minimal. During this phase, the front strengthens and restratifies, whereas overturning cells induce large advective fluxes. Heat and oxygen are efficiently transported along isopycnals, with fluxes up to −40 W m^−2^ and −20 mmol m^−2^ day^−1^, respectively. In contrast, mixing rates, while still elevated in the frontal zone, are substantially lower than during the wind-forced phase (Fig. 3), resulting in weak turbulent fluxes. Overall, advective fluxes exceed turbulent fluxes by roughly an order of magnitude (Fig. 2).

In phase 3, beginning on 3 July around 18:00 UTC, upwelling-favorable winds intensify and gain an inertial component, driving the front offshore and inducing oscillations at the local inertial frequency (Fig. 2, A and C). The freshwater front becomes shallower, and signatures of SI vanish. As discussed above, this apparent disappearance may reflect a dynamical transition or changes in spatial sampling as the measurements no longer capture the full extent of the front. Note that Qu *et al.* (*40*), using SUNRISE data and numerical models, demonstrate that interactions between inertial currents and a front can also facilitate vertical exchange in the northern Gulf of Mexico. This exchange is driven by inertial motions and is fundamentally different from the instability-driven mechanism examined in the present study. With the present observations, however, it is not possible to assess how this inertially driven process may interact with SI as the full frontal structure is not resolved.

## DISCUSSION

Our observations reveal remarkably clear and persistent signals of SI at a freshwater front, lasting nearly 4 days. The instabilities spanned the entire water column, connecting surface and bottom waters. The event evolved across two distinct forcing regimes, with different implications for vertical exchange, transitioning from a regime where both turbulence and advection contribute, to one where advection governs vertical exchange.

**Fig. 5.**
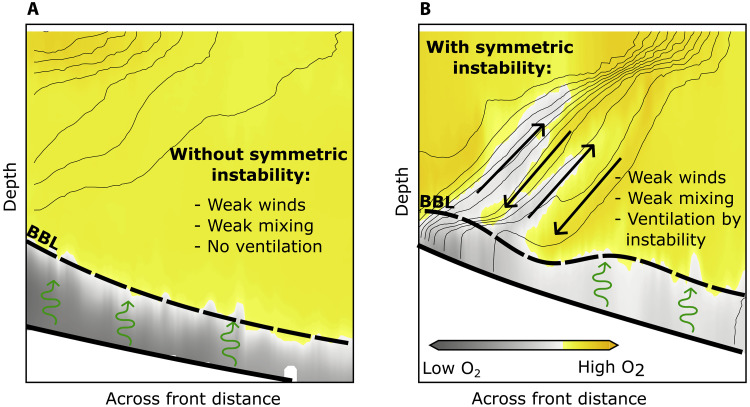
Impact of SI on vertical exchange. Schematic sections illustrating the role of SI for the vertical oxygen flux. Panels show two cross-frontal oxygen sections measured under similar weak wind conditions: (**A**) before instability event (22 June 2021) and (**B**) during the event (3 July 2021). The dashed black line marks the extent of the BBL, and thin black lines indicate isopycnals. Green curly arrows indicate turbulent mixing, whereas black straight arrows denote advection. Because of weak surface forcing and mixing, vertical exchange is absent in (A), resulting in poorly ventilated bottom waters. In contrast, (B) shows the enhanced ventilation of deeper waters during the instability event, driven by slantwise overturning cells.

An intriguing aspect of our results is that the signals of SI persists for at least 40 hours after destabilizing wind forcing ceases. The observed longevity of the SI signals is likely influenced by the strong stratification in the frontal region (*29*). After the destabilizing wind forcing relaxes, coherent slantwise overturning cells continue to drive vertical advection of heat and oxygen, and strong advective fluxes are observed. This impact of SI for vertical exchange is illustrated in Fig. 5, which compares two cross-front sections sampled under similarly weak wind forcing conditions. Before the instability developed (Fig. 5A), vertical exchange is minimal in the absence of mechanisms that can break the stratification barrier. In contrast, during phase 2 (Fig. 5B), SI overturning cells sustain vertical transport, despite weak winds and mixing, demonstrating how SI can drive vertical exchange in an otherwise stably stratified coastal environment.

It is important to consider whether the SI-driven vertical exchange mechanism observed here is relevant at larger space and timescales. Although the observed vertical exchange was confined to a narrow cross-front zone of about 10 to 15 km, it likely extended much farther in the along-front direction. On the basis of the duration of the event and the advection speed past the sampling site, we estimate that SI was active along at least a 50-km stretch of the front. This implies that SI, although spatially localized across the front, exerts a broader influence along the shelf. The associated vertical oxygen fluxes are also substantial: For example, a flux of 20 mmol m^−2^ day^−1^ converging into a BBL of ~5-m thickness would correspond to an oxygen increase of about 4 mmol m^−3^ day^−1^, which is on the same order as respiration rates on the shelf during developing hypoxia (*9*). Such fluxes could therefore slow or prevent the onset of hypoxia in affected regions. Notably, the location of the observed event lies near the southern edge of the seasonal hypoxic zone, where the frequency and severity of hypoxia typically decline [see maps of hypoxia frequency in (*9*, *41*)]. Although causality remains speculative, the presence of a sustained vertical exchange mechanism like SI may contribute to this spatial gradient in oxygenation.

SI may be an underappreciated mode of vertical transport in the northern Gulf of Mexico. Although this study documents a single event, it points to the potential for SI to influence regional tracer budgets and modulate biogeochemical conditions on larger scales. This is particularly relevant for this region, where seasonal bottom-water hypoxia is widespread and primarily linked to strong stratification and weak vertical mixing (*7*–*9*). The instability-driven ventilation pathway is likely most relevant during summer, when winds are often too weak for their associated mixing to terminate hypoxic conditions. Under these conditions, SI provides an energetically efficient pathway for vertical exchange that can shape bottom-water oxygen dynamics. Understanding the factors that control heat content in this region is also critical for improving forecasts of hurricane intensity and development (*4*, *6*). These results call for further efforts to evaluate the climatological importance of SI and its role in shaping heat and oxygen dynamics in the northern Gulf of Mexico.

Most regional and global ocean models must parameterize submesoscale dynamics. Our results show that, during weak or stabilizing surface forcing conditions, advective transport associated with SI exceeds turbulent fluxes by an order of magnitude. Achieving a similar vertical redistribution through the observed mixing rates alone would require more than 2 weeks of mixing, underlining the efficiency and potential ecological relevance of SI-driven transport. Although existing parameterizations focus on representing the dissipative and restratifying effects of SI (*42*, *43*), our observations suggest that the advective component of SI tracer transport should be incorporated into numerical models, particularly those aiming to simulate biogeochemical processes.

Our findings from the northern Gulf of Mexico point to an instability-driven vertical exchange mechanism that may play an important role in coastal oceans more broadly. Although the event occurred under specific atmospheric and oceanic conditions, similar combination of forcing and stratification regimes are not unique to this region and may arise in other coastal systems. Buoyant river plumes around the world flow over relatively shallow continental shelves and are subject to instability-favorable wind forcing (*44*–*48*). Similar physical conditions—strong surface fronts and instability-favorable winds—also occur in coastal upwelling zones, making them favorable environments for SI as well (*33*, *49*). As a potentially widespread and underappreciated mechanism, SI could facilitate vertical exchange of heat, oxygen, nutrients, carbon, and other tracers. The relevance of the mechanism therefore needs to be evaluated across a broader range of coastal environments.

## MATERIALS AND METHODS

### The SUNRISE campaign

The SUNRISE 2021 field campaign (20 June to 9 July) took place on the Louisiana shelf, a region shaped by buoyant river plumes and energetic submesoscale dynamics. The campaign aimed to investigate how wind-driven near-inertial motions interact with fresh water–induced density fronts. To resolve the evolving three-dimensional structure of these features, the campaign was conducted with two research vessels: *R/V Pelican* and *R/V Walton Smith*. The vessels often coordinated sampling by conducting parallel cross-front sections spaced 1 km apart. The two-ship approach enabled synoptic sampling of horizontal and vertical gradients and provided an unprecedented view of frontal processes with high spatial and temporal resolution.

Our study focuses on a subset of the SUNRISE 2021 observations collected between 29 June and 4 July 2021. During a 5-day period, 30 cross-front transects were conducted, many of them sampled in parallel by both vessels. On average, each transect required ~3.5 hours to complete. Note that the full campaign encompassed a range of forcing regimes [see the supplement material of Qu *et al.* (*40*) for an overview], and although the primary goal of the campaign was to investigate interactions between near-inertial motions and density fronts, inertial forcing was relatively weak during the period analyzed here.

To characterize velocity structure, we use data from 1200-kHz ADCPs on both ships. These ADCPs provide high-resolution profiles (0.5 m) and are capable of resolving vertical shear in frontal regions. In waters deeper than ~20 m, the range of the 1200-kHz ADCP is insufficient to reach the bottom, and we fill these gaps using data from a 600-kHz ADCP interpolated onto the vertical grid of the 1200-kHz profiles. The ADCP data are averaged into 60-s bins. At an average ship speed of 2.7 knots during sampling, this corresponds to a horizontal resolution of ~80 m.

Hydrographic structure was measured using a Rockland Scientific VMP250 vertical microstructure profiler (VMP) and RBR Concerto CTD with an attached Rinko dissolved oxygen sensor. While conducting the cross-front sections on both ships, profiling was done continuously with either the VMP or the CTD, resulting in profiles approximately every 100 m. Most data were collected with the VMP, whereas the CTD was only used for a limited number of sections. In total, across both vessels, 8410 VMP and 1595 CTD profiles were collected during the 5-day period analyzed in this study. Dissipation rates of turbulent kinetic energy were derived from the VMP shear microstructure measurements, with profiling extending to the bottom using crash guards. The VMP is equipped with two shear probes sampling at 512 Hz to resolve small-scale velocity gradients. Turbulent kinetic energy dissipation, ϵ, is estimated from shear variance over a defined segment length, using spectral methods (*39*, *50*–*52*). To ensure sufficient resolution and data quality, profiles were collected at fall rates of ~1 m s^−1^.

### Definition of the front

To determine the location of the front, we define a salinity threshold, identifying it as the northernmost latitude where absolute salinity exceeds 35.3 g kg^−1^. Surface front positions are based on salinity averaged over the upper 1 m, whereas bottom front positions are derived from salinity averaged over the bottom 1 m. Front location is computed separately for each ship.

The orientation of the front is estimated using two methods. The first method is based on the horizontal buoyancy gradient, calculated from data collected by both ships (see the section below). We vertically average the buoyancy gradient over the upper 2 m and compute the orientation by taking a magnitude-weighted mean of its direction. To suppress noise from small-scale variability, particularly inshore of the front, we average within a ±2.5-km window around the location of the surface front.

The second method relies on velocity data to estimate front orientation. The direction of the front is determined by calculating the speed-weighted average of the velocity direction. Because the shallowest velocity measurements are at a 4.5-m depth, the front location is identified between 4.5 and 5 m depth, considering data within 0 to −5 km relative to the surface front position. The speed-weighted average approach provides an estimate of the front orientation for the section where only one ship were present. However, velocity measurements are not available at the surface, and in some cases, the estimated front location at a 4.5- to 5-m depth falls outside the measured section.

For sections where both ships were present, we find that both methods for estimating frontal orientation agree well. To get a single orientation for these sections, we average the results of both methods. In three sections, no velocity-based estimate or buoyancy-based estimate was possible, and for these cases, the front orientation was interpolated in time.

### Calculation of variables based on data of one ship and two ships

We require velocity and buoyancy gradient estimates, in both horizontal and vertical directions, to calculate higher-order quantities (e.g., Ertel PV). We calculate these gradients slightly differently for times when just one ship is present and when two ships sample in parallel. We call these approaches the one-ship and two-ship solution.

When both ships sample in parallel, horizontal derivatives can be estimated using a plane-fitting approach (*53*). To achieve this, we define an artificial track between the two ships and compute the derivatives at each point along this track by fitting a linear function of the form: $u=u_{0}+u_{x}x+u_{y}y$, where subscripts denote derivatives in the along (*x*) and across (*y*) front direction (Fig. 1). The fitting procedure considers data within a defined influence region around each point on the artificial track. To ensure a consistent spatial weighting of data points, we define an elliptical influence area with a semimajor axis of 2 km and a semiminor axis of $\sqrt{2}$ km (fig. S6). The ellipse is oriented with its long axis perpendicular to the artificial track to optimize the estimation of horizontal gradients. To get the vertical derivative along the artificial track, we define, at each point on the artificial track, a perpendicular line and select the four closest measurement points from each ship. The vertical derivative along the track is then estimated by averaging the vertical derivatives at these selected points (fig. S6).

Using this approach, we calculate the horizontal and vertical derivatives of the along-front (*u*) and across-front (*v*) velocity components as well as the derivatives for the buoyancy ($b=-g\rho /\rho_{0}$; *g* the acceleration due to gravity and ρ is the density). We can then calculate fields of vorticity ($\zeta =v_{x}-u_{y}$) and PV [$q=\left(\zeta +f\right)b_{z}+b_{y}u_{z}-b_{x}v_{z}$].

When only one ship is present, we assume that the variability in the across-front direction is larger than in the along-front direction. The vorticity then becomes $\zeta =-u_{y}$, and the PV $q=\left(\zeta +f\right)b_{z}+b_{y}u_{z}$. Derivatives are calculated using a linear fit, rather than a plane fit. We choose an along section window of 1 km for the linear fit, which matches roughly the along section influence radius of the two-ship method.

For sections where both ships were present, we compare the one-ship and two-ship solutions to assess the reliability of the one-ship approach (see fig. S7 for some exemplary comparisons). Overall, there is excellent agreement between one-ship and two-ship solutions. This agreement reflects the dominance of variability in the across-front direction, which is resolved by the one-ship sections.

### Bottom stress

To estimate bottom stress, we use turbulence profiles from the VMP that hit the bottom. The BBL is first identified using a density-based criterion: We define the BBL as the region extending from the seafloor to the first depth level where the density exceeds the near-bottom value by more than 0.01 kg m^−3^. Within this layer, bottom stress τ_b_ is estimated following (*50*, *54*) as $\tau_{b}=\rho u_{*}^{2}$, where ρ is the local density and $u_{*}$ is the friction velocity. The friction velocity is computed from turbulence measurements as $u_{*}={\langle \epsilon \kappa z^{\prime }\rangle }^{1/3}$, where ϵ(z) is the dissipation rate of turbulent kinetic energy, $z^{\prime }$ is the height above bottom, and κ=0.4 is the von Kármán constant. To determine the direction of the bottom stress vector, we use velocity measurements from the ADCP and assume that the stress acts opposite to the flow direction immediately above the BBL as bottom stress decelerates the flow, which can be expressed as an acceleration in the opposing direction.

### Buoyancy fluxes

To quantify the destabilizing or restratifying effect of wind stress acting on lateral buoyancy gradients, we calculate the EBF (*27*, *34*, *40*). The EBF is defined as

$${\text{EBF}}_{\text{surf}}=M_{E}\cdot \nabla_{h}b$$ (1)

where *M*_E_ is the Ekman transport and $\nabla_{h}b$ is the horizontal buoyancy gradient. The Ekman transport can be calculated assuming either a steady or time-dependent response to wind stress. The steady case assumes a constant wind stress over time, and the Ekman transport then simply depends on the wind stress, the Coriolis parameter, and a reference density (*18*). Because wind stress varies substantially over the course of our observations (Fig. 2A), we instead calculate the time-dependent Ekman transport using a slab model (*40*). Our starting point is the mixed-layer momentum equations

$$\frac{{\mathrm{dM}}_{x}}{\mathrm{dt}}-{\mathrm{fM}}_{y}=\frac{\tau_{x}}{\rho }-{\mathrm{rM}}_{x}$$ (2)

$$\frac{{\mathrm{dM}}_{y}}{\mathrm{dt}}+{\mathrm{fM}}_{x}=\frac{\tau_{y}}{\rho }-{\mathrm{rM}}_{y}$$ (3)

where τ*_x_* and τ*_y_* is the along-front and across-front wind stress, *r* is a damping coefficient, and $\left(M_{x},M_{y}\right)$ is the time-dependent Ekman transport. As an initial condition, we choose $M_{x}=M_{y}=0$. To reduce the influence of the initial conditions, we start the integration on 23 June 2021 and thus about a week before the event. For the damping term, we use r=0.1f (*55*). To then calculate the EBF, we multiply the Ekman transport with the horizontal buoyancy gradient (Eq. 1).

We also calculate the EBF in the BBL. Unfortunately, we do not have a full time series of the bottom stress as we only have data during the VMP profiling. Thus, we cannot calculate the time-dependent Ekman transport in the BBL. Instead, we calculate the steady solution

$${\text{EBF}}_{\text{BBL}}=\frac{\tau_{x}^{b}}{\rho_{0}f}\frac{\text{∂}b}{\text{∂}y}$$ (4)

where $\tau_{x}^{b}$ is the bottom stress in the along-front direction, and the buoyancy gradient is averaged over the lowermost 2 m of the water column. Note the sign convention for the bottom EBF, reflecting the direction of Ekman transport the BBL (*38*).

Surface heat fluxes can both stabilize and destabilize the water column, depending on their direction and magnitude. Net surface heat fluxes (*Q*_net_) are estimated using the COARE algorithm (*56*, *57*), applied to meteorological measurements from shipboard sensors with a temporal resolution of 60 s. The total surface buoyancy flux is then computed as

$$B=-\frac{g\alpha }{\rho_{0}c_{p}}Q_{\text{net}}+g\beta S_{\text{flux}}$$ (5)

where the first term represents the buoyancy flux due to heat exchange, *g* is the gravitational acceleration, $\alpha =2\times 10^{-4}\;K^{-1}$ is the thermal expansion coefficient, $\rho_{0}=1025\;\text{kg}\;m^{-3}$ is the reference density of seawater, and $c_{p}=3985\;J\;{\text{kg}}^{-1}\;K^{-1}$ is the specific heat capacity of seawater. 

The second term represents the buoyancy flux associated with freshwater forcing. Salt flux is computed as $S_{\text{flux}}=S\left(E-P\right)$, where *S* is the surface salinity, and *E* is the evaporation derived from latent heat flux (LHF) via: $E=-\frac{\text{LHF}}{\rho_{w}L_{v}}$ with $\rho_{w}=1000\;\text{kg}\;m^{-3}$ the density of fresh water and $L_{v}=2.5\times 10^{6}\;J\;{\text{kg}}^{-1}$ the latent heat of vaporization. Precipitation (*P*) is obtained from onboard rain sensors. The saline contraction coefficient is $\beta =7.6\times 10^{-4}\;\text{kg}\;g^{-1}$.

### Vertical oxygen and heat fluxes

To quantify the vertical exchange of heat and oxygen during the SI event, we estimate both vertical turbulent and advective fluxes within the frontal zone.

Turbulent fluxes are derived from VMP profiles of dissipation rate (ϵ) and buoyancy frequency (*N*^2^). We calculate the eddy diffusivity as $K_{\rho }=\Gamma \epsilon N^{-2}$ (*39*), assuming a flux coefficient of Γ=0.2 (*58*). The resulting turbulent fluxes of temperature and oxygen are computed as $F_{\text{turb}}=-K_{\rho }\text{∂}T/\text{∂}z$ and $F_{\text{turb}}=-K_{\rho }\text{∂}O_{2}/\text{∂}z$, respectively. To focus on the stratified frontal region, fluxes are only computed where $N^{2}\geq 2.5\times 10^{-4}\;s^{-2}$.

To compute the advective fluxes we first have to estimate the vertical velocities. For that, we introduce a streamfunction, ψ, describing the baroclinic part of the across-front velocity and the vertical velocity

$$v_{\text{bc}}=v-\frac{1}{H}\int_{-H}^{0}vdz=\frac{\text{∂}\psi }{\text{∂}z},\;w=-\frac{\text{∂}\psi }{\text{∂}y}$$ (6)

where *H*(*y*) is the ocean depth and, as with the one-ship solution, we are assuming that the across-front variability dominates and that the vertical velocity of the overturning motions can be computed from continuity in the *y*-*z* plane. Considering only the baroclinic part of the flow and imposing ψ = 0 on both the sea surface and seafloor neglects any vertical motion associated with the free surface. However, using both boundaries in this way is advantageous when working with observations as there are now two points where the streamfunction is known exactly, which reduces the accumulation of error when integrating in the vertical. The streamfunction is given by

$$\begin{array}{c} \psi \left(y,z\right)=\int_{-H}^{z}v_{\text{bc}}\left(y,z^{\prime }\right)dz^{\prime }\\ =\int_{-H}^{z}vdz^{\prime }-\frac{H+z}{H}\int_{-H}^{0}v\;dz^{\prime }=\frac{-z}{H}\int_{-H}^{z}vdz^{\prime }-\frac{H+z}{H}\int_{z}^{0}vdz^{\prime } \end{array}$$ (7)

The final expression can be recognized as a particular weighted average of the streamfunctions computed by integrating the full velocity up from the seafloor and down from the sea surface. These integrals are evaluated numerically with missing values near the boundaries filled using nearest neighbor interpolation.

The vertical velocity is then computed by evaluating the horizontal derivative. However, *w* is a very noisy field—both for physical reasons, e.g., the presence of high-frequency waves, and due to observational uncertainty. Therefore, we choose to compute a smoothed *w* using a Gaussian filter

$$\begin{array}{c} w=-\int \frac{\text{∂}\psi }{\text{∂}y^{\prime }}\frac{1}{L_{s}\sqrt{2\pi }}\text{exp}\left(-\frac{{\left(y-y^{\prime }\right)}^{2}}{2L_{s}^{2}}\right)dy^{\prime }\\ =\int \psi \left(y^{\prime }\right)\frac{\left(y-y^{\prime }\right)}{L_{s}^{3}\sqrt{2\pi }}\text{exp}\left(-\frac{{\left(y-y^{\prime }\right)}^{2}}{2L_{s}^{2}}\right)dy^{\prime } \end{array}$$ (8)

with the second integral evaluated numerically. The smoothing length scale *L*_s_ = 500 m was chosen to be short compared to the *O*(4 km) wavelength of the SI to ensure a faithful representation of the vertical velocity associated with the overturning cells. It should be noted that this method of computing the horizontal derivative smooths the resulting field more aggressively than the linear fits used by the one-ship and two-ship solutions.

Examples of this approach are shown in fig. S8, which displays the measured across-front velocity, the derived streamfunction ψ, and the resulting vertical velocity. The inferred vertical velocity field exhibits alternating upwelling and downwelling patterns that are consistent with overturning cells associated with SI.

With an estimate of the vertical velocity in hand, we compute the vertical advective fluxes of heat and oxygen. The advective fluxes are defined as $F_{\text{adv}}=w^{\prime }T^{\prime }$ for temperature and $F_{\text{adv}}=w^{\prime }{O_{2}}^{\prime }$ for oxygen, where primes denote anomalies relative to the mean vertical profiles. Mean profiles are calculated separately for each section using data within a cross-front distance of −15 to 5 km from the surface front. To obtain the time series of vertical advective fluxes shown in Fig. 2, the fluxes are averaged over the frontal region, defined as locations where $N^{2}\geq 2.5\times 10^{-4}\;s^{-2}$, consistent with the criterion used for the turbulent flux estimates.
