# Supplementary material for: Symmetric instability drives exchange between surface and bottom waters in a coastal front
Source: Sci Adv. 2026 May 8;12(19):eaeb9841. doi: 10.1126/sciadv.aeb9841 (PMC13155336; doi:10.1126/sciadv.aeb9841)
Supplement: Supplementary file 1 — Supplementary Text Figs. S1 to S8 [file sciadv.aeb9841_sm.pdf]

Science Advances
AAAS

# Supplementary Materials for

## Symmetric instability drives exchange between surface and bottom waters in a coastal front

Mareike Körner *et al.*

Corresponding author: Mareike Körner, mareike.koerner@oregonstate.edu

*Sci. Adv.* **12**, eaeb9841 (2026)
DOI: 10.1126/sciadv.aeb9841

**This PDF file includes:**

Supplementary Text
Figs. S1 to S8

# Supplementary Text

## Instability classification

Negative Ertel potential vorticity (PV) is a necessary condition for symmetric instability (SI) in the northern hemisphere (*20, 23*), but it is not sufficient to uniquely identify SI, as other forms of instability, such as gravitational or inertial, can also occur when PV is negative. To distinguish among these, we compute the instability angle (*21*), a diagnostic that identifies which term in the PV equation dominates and the primary energy source of the instability. The instability angle is based on the balanced Richardson number, defined as:

$$\mathrm{Ri_B} = \frac{N^2}{\left(\frac{\partial u_g}{\partial z}\right)^2} = \frac{f^2 N^2}{|\nabla_h b|^2}, \tag{S1}$$

where $N^2$ is the squared buoyancy frequency, $\frac{\partial u_g}{\partial z}$ is the vertical shear of the geostrophic velocity, $f$ is the Coriolis parameter, and $|\nabla_h b|^2$ is the squared horizontal buoyancy gradient. The angle is then defined as

$$\phi_{\mathrm{Ri_B}} = \tan^{-1}\left(\frac{-|\nabla_h b|^2}{f^2 N^2}\right), \tag{S2}$$

which expresses the ratio of stabilizing stratification to destabilizing vertical shear in a geostrophic flow. Instability occurs when the instability angle falls below a critical threshold,

$$\phi_{\mathrm{Ri_B}} < \phi_c \equiv \tan^{-1}\left(-\frac{\zeta_g}{f}\right), \tag{S3}$$

where $\zeta_g$ is vertical component of the absolute vorticity of the geostrophic flow. For $\phi_{\mathrm{Ri_B}} < \phi_c$ we can then differentiate between gravitational, symmetric, and inertial instabilities. Details on the classification of instability types and the corresponding angle ranges for cyclonic and anticyclonic flows can be found in Thomas et al. (*21*).

Importantly, the method assumes leading-order geostrophic balance. We verify that this assumption holds in our case by evaluating the thermal wind balance (Fig. 4), supporting the validity of using the instability angle to classify the observed flow instabilities.

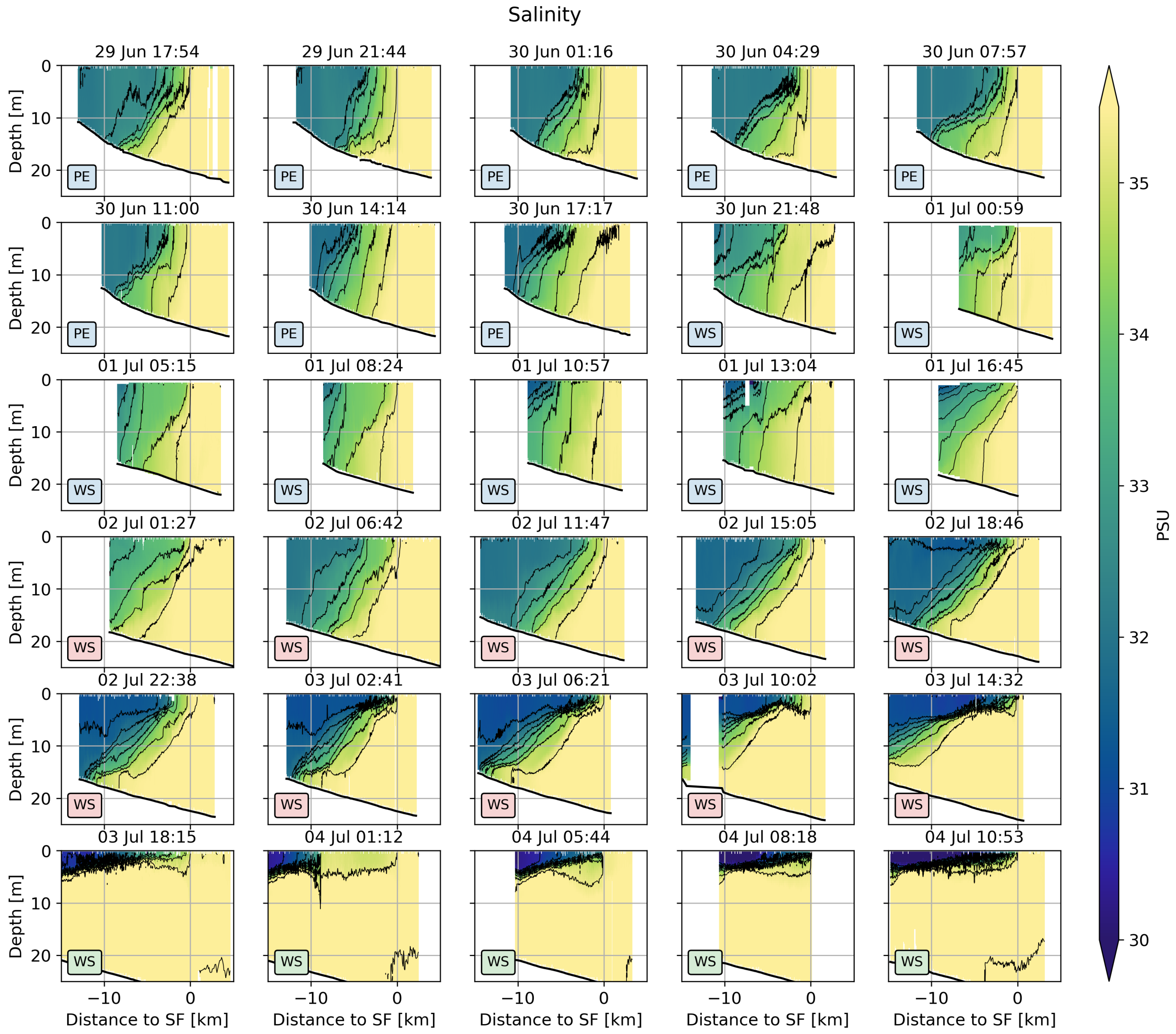


**Figure S1**: **Temporal evolution of salinity across the front.** Cross-front sections of salinity as a function of depth and distance from the surface front. The time in each panel title indicates the start of the corresponding section measurement. Thin black contours represent isopycnals. The text box in the lower left corner of each panel indicates the sampling vessel, *R/V Walton Smith* (WS) or *R/V Pelican* (PE), and its background color denotes the observational phase, as defined in Fig. 2.

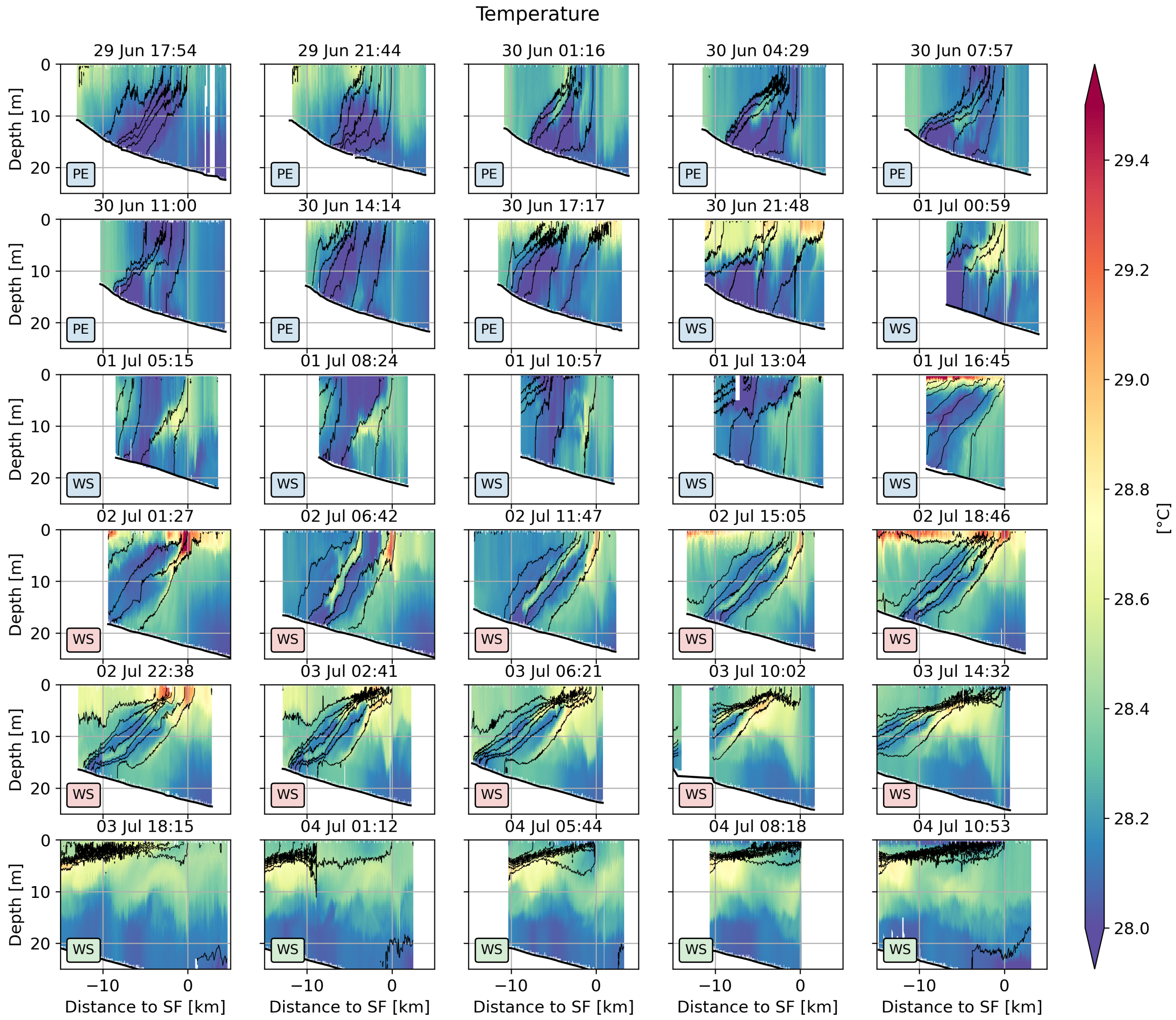


**Figure S2**: **Temporal evolution of temperature across the front.** Cross-front sections of temperature as a function of depth and distance from the surface front. The time in each panel title indicates the start of the corresponding section measurement. Thin black contours represent isopycnals. The text box in the lower left corner of each panel indicates the sampling vessel, *R/V Walton Smith* (WS) or *R/V Pelican* (PE), and its background color denotes the observational phase, as defined in Fig. 2.

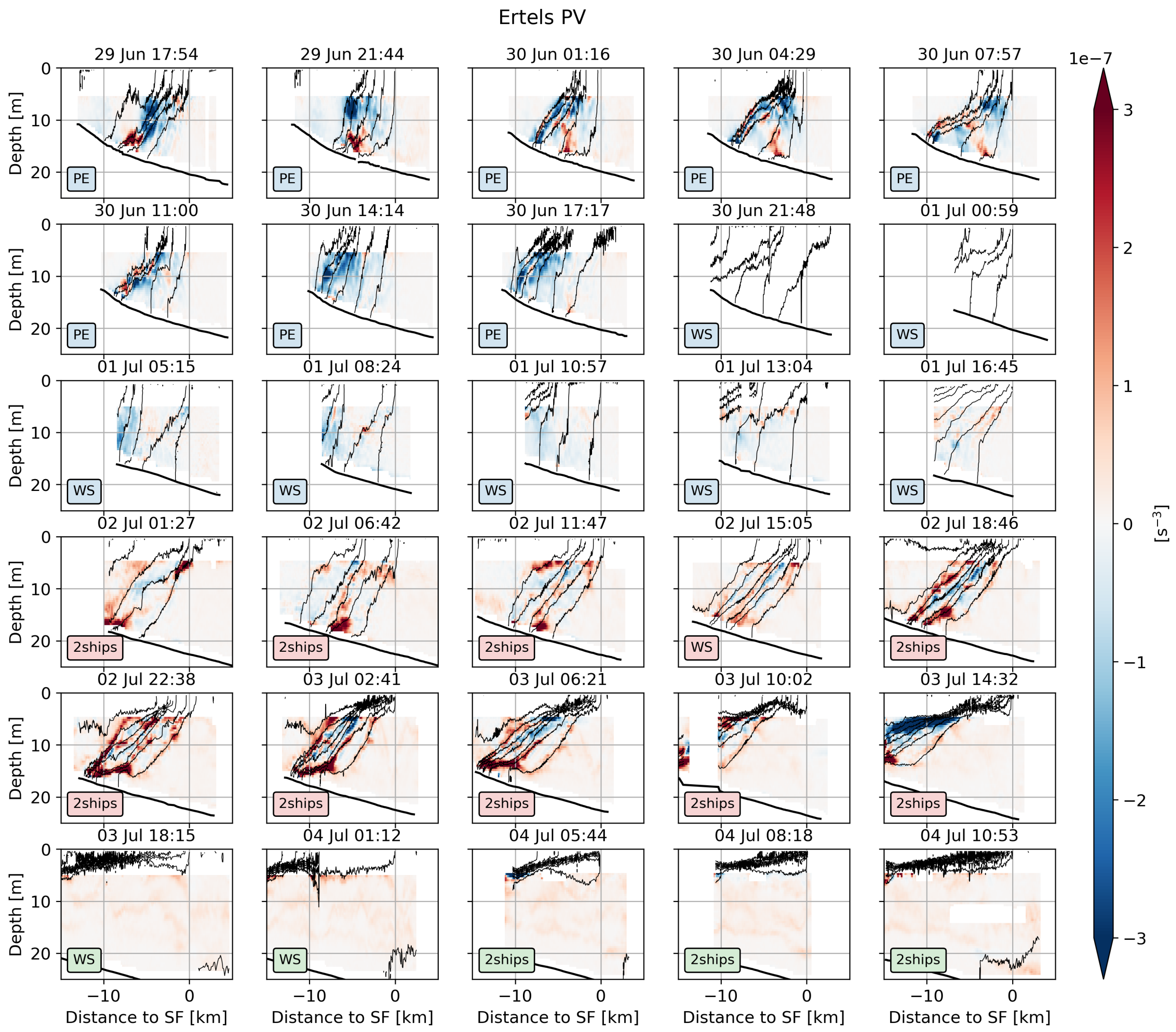


**Figure S3**: **Cross-front sections of Ertel potential vorticity.** Cross-front sections of Ertel potential vorticity as a function of depth and distance from the surface front. The time in each panel title indicates the start of the corresponding section measurement. Thin black contours represent isopycnals. The text box in the lower left corner of each panel indicates the sampling vessel, *R/V Walton Smith* (WS) or *R/V Pelican* (PE), or “2ships” when the calculation uses velocity data from both ships. The background color of the text box denotes the observational phase, as defined in Fig. 2.

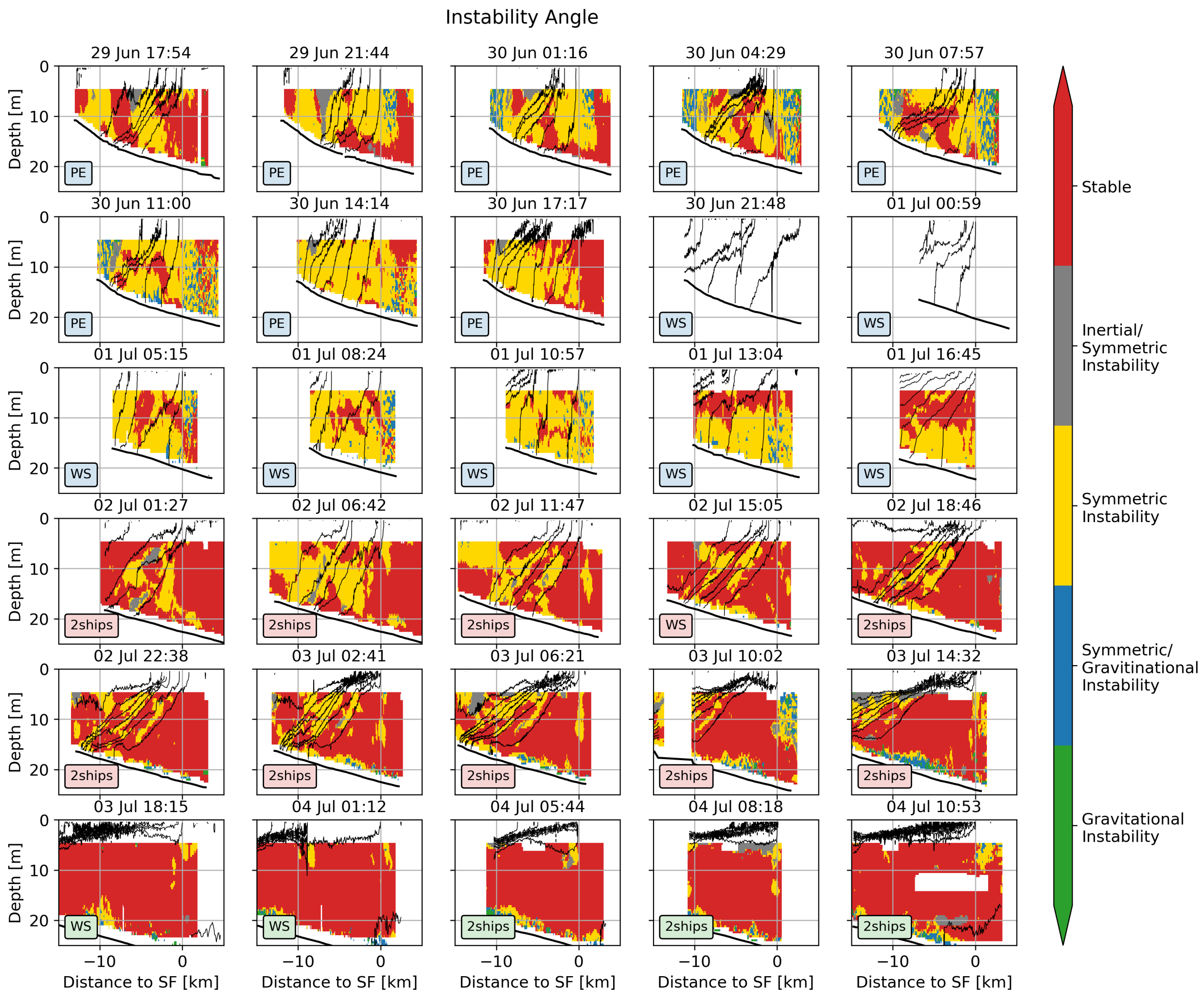


**Figure S4**: **Cross-front sections of instability angle.** Cross-front sections of instability angle as a function of depth and distance from the surface front. The time in each panel title indicates the start of the corresponding section measurement. Thin black contours represent isopycnals. The text box in the lower left corner of each panel indicates the sampling vessel, *R/V Walton Smith* (WS) or *R/V Pelican* (PE), or "2ships" when the calculation uses data from both ships. The background color of the text box denotes the observational phase, as defined in Fig. 2.

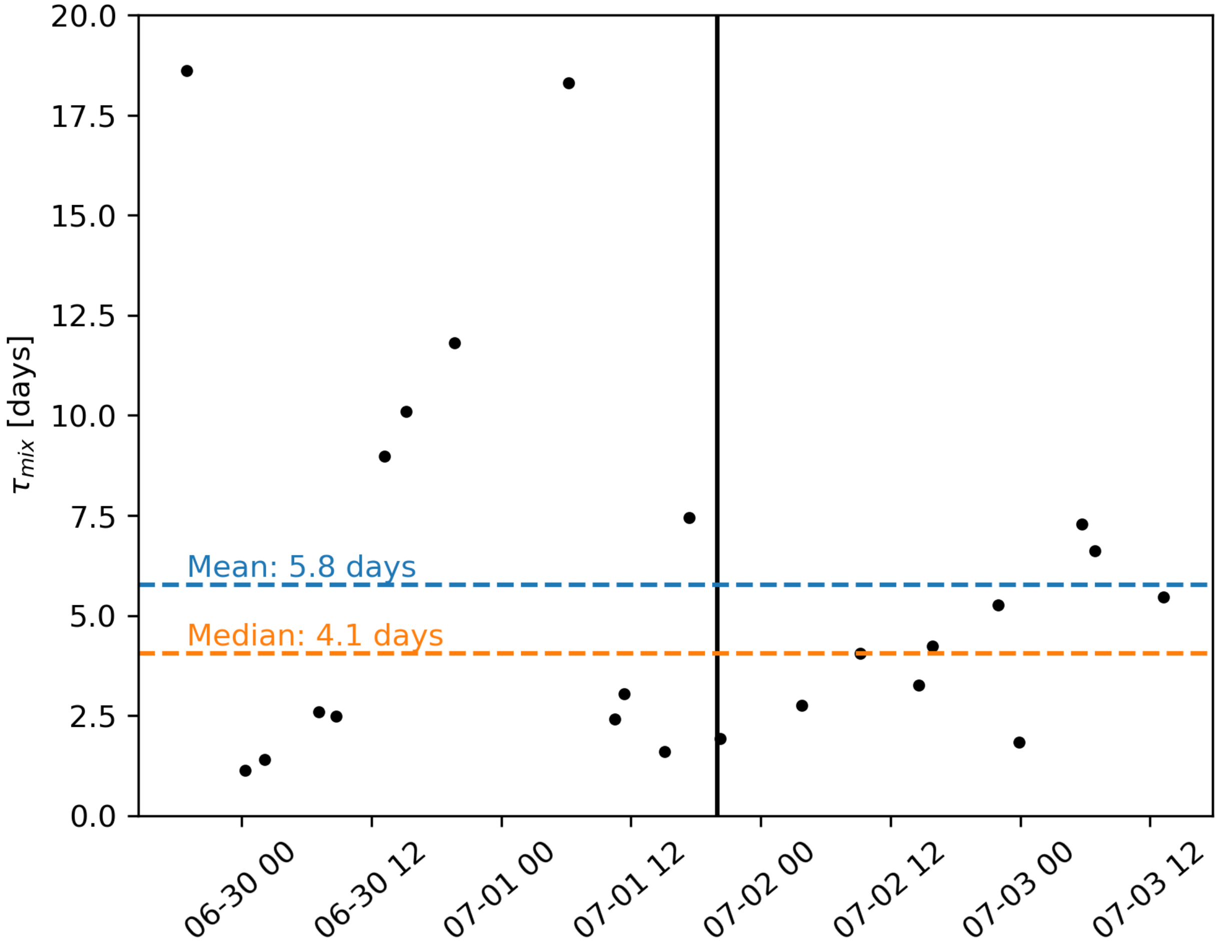


**Figure S5**: **Theoretical time scale for symmetric instability** The theoretical time scale $\tau_{mix}$ for SI-driven fluxes to erode the thermal wind balance, calculated following Wienkers et al. (*29*) (their equation 5.5). $\tau_{mix}$ is calculated for each section individually (black dots). The dashed blue and orange lines indicate the mean (5.8 days) and median (4.1) mixing timescales, respectively. The vertical black line separates Phase 1 and Phase 2 of the observational periods.

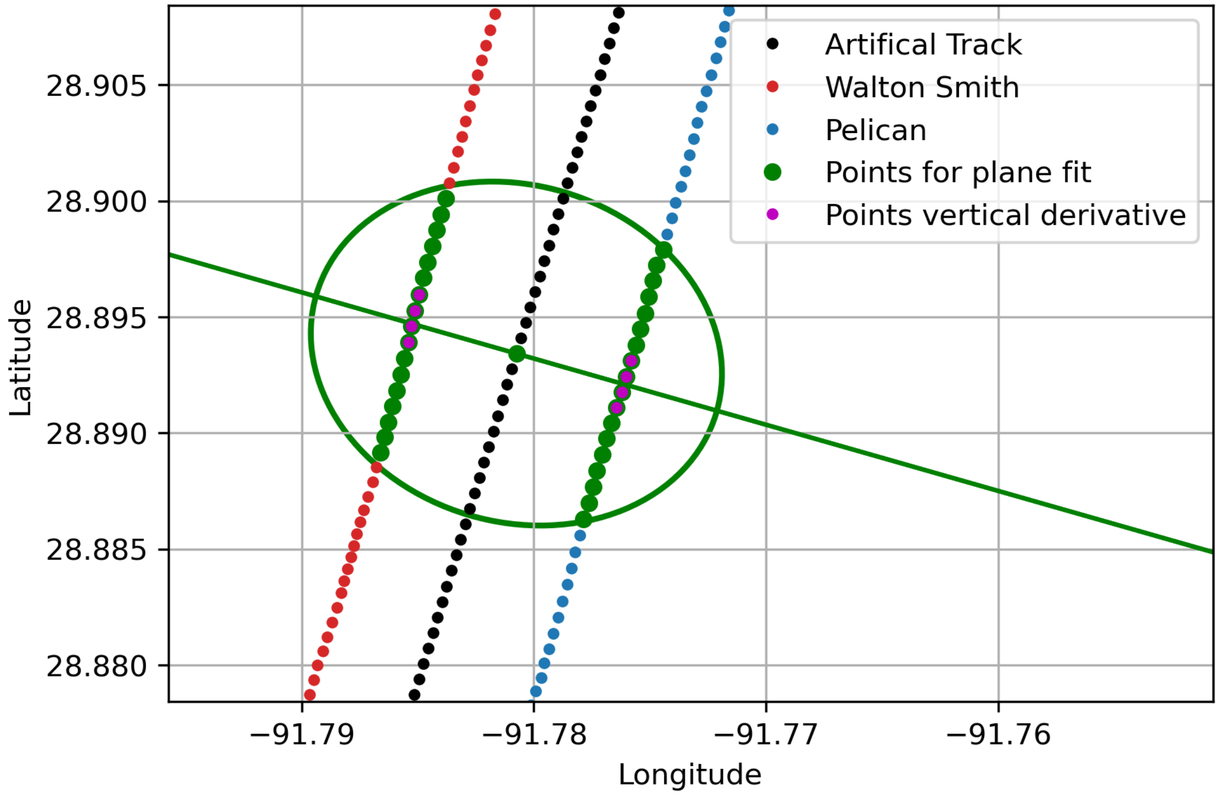


**Figure S6**: **Method for calculating horizontal and vertical derivatives from two-ship solution** Dotted line mark velocity data points from the *R/V Walton Smith* (red) and *R/V Pelican* (blue), and the black line indicates the artificial track between the ships. Green dots mark the data points used for horizontal gradient estimation via plane fitting within an elliptical influence region (green ellipse). Green line shows the cross-track orientation. Magenta dots highlight the eight points (four from each ship) used to average the vertical derivative for the artificial track.

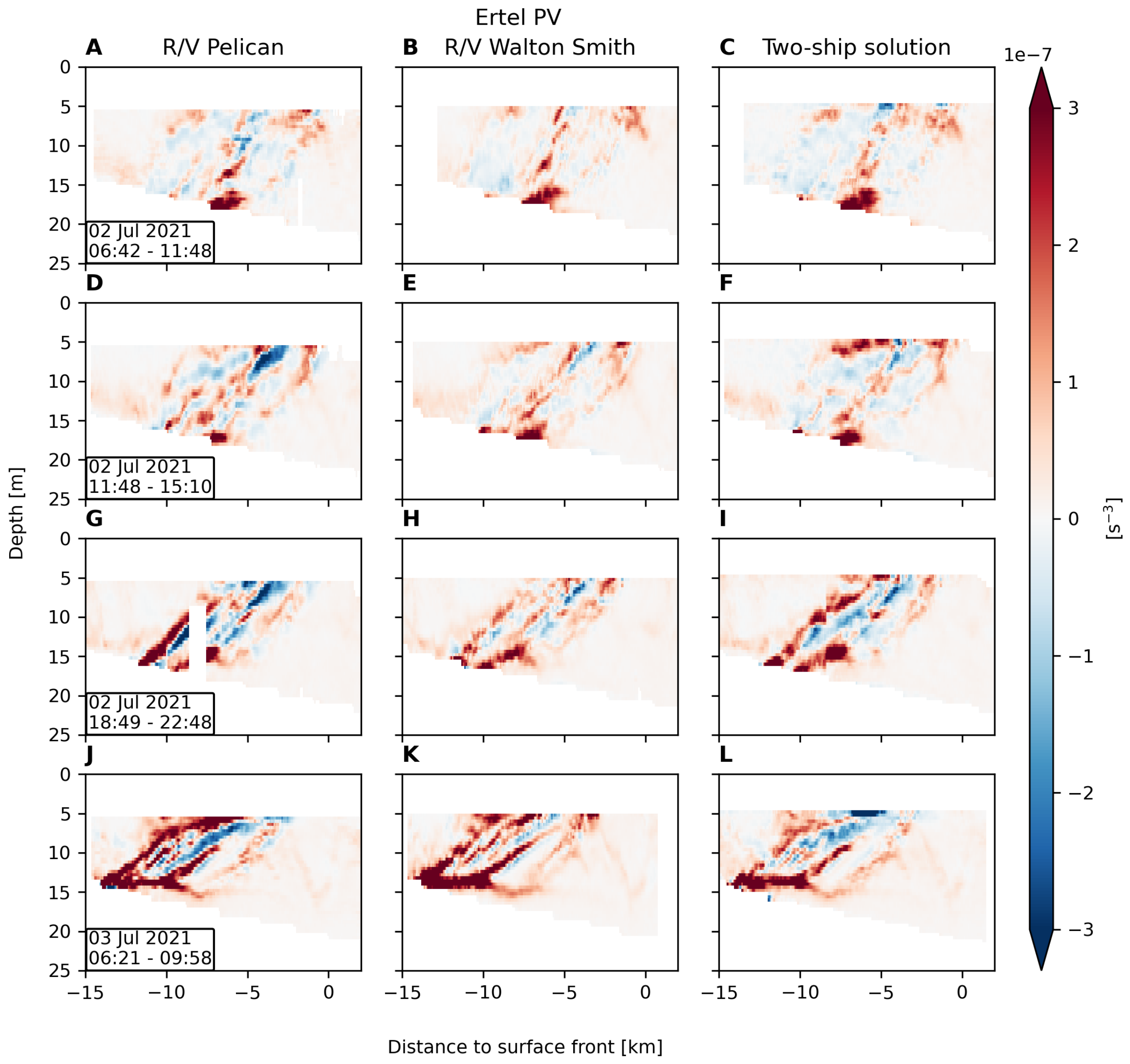


**Figure S7**: **Ertel Potential Vorticity (PV) estimated using one-ship and two-ship approaches.** The left and middle columns show PV calculated from the one-ship solutions using data from *R/V Pelican* and *R/V Walton Smith*, respectively. The right column presents PV computed using the two-ship method. Each row corresponds to a distinct cross-front section, with the date and time range (UTC) indicated in the leftmost panel.

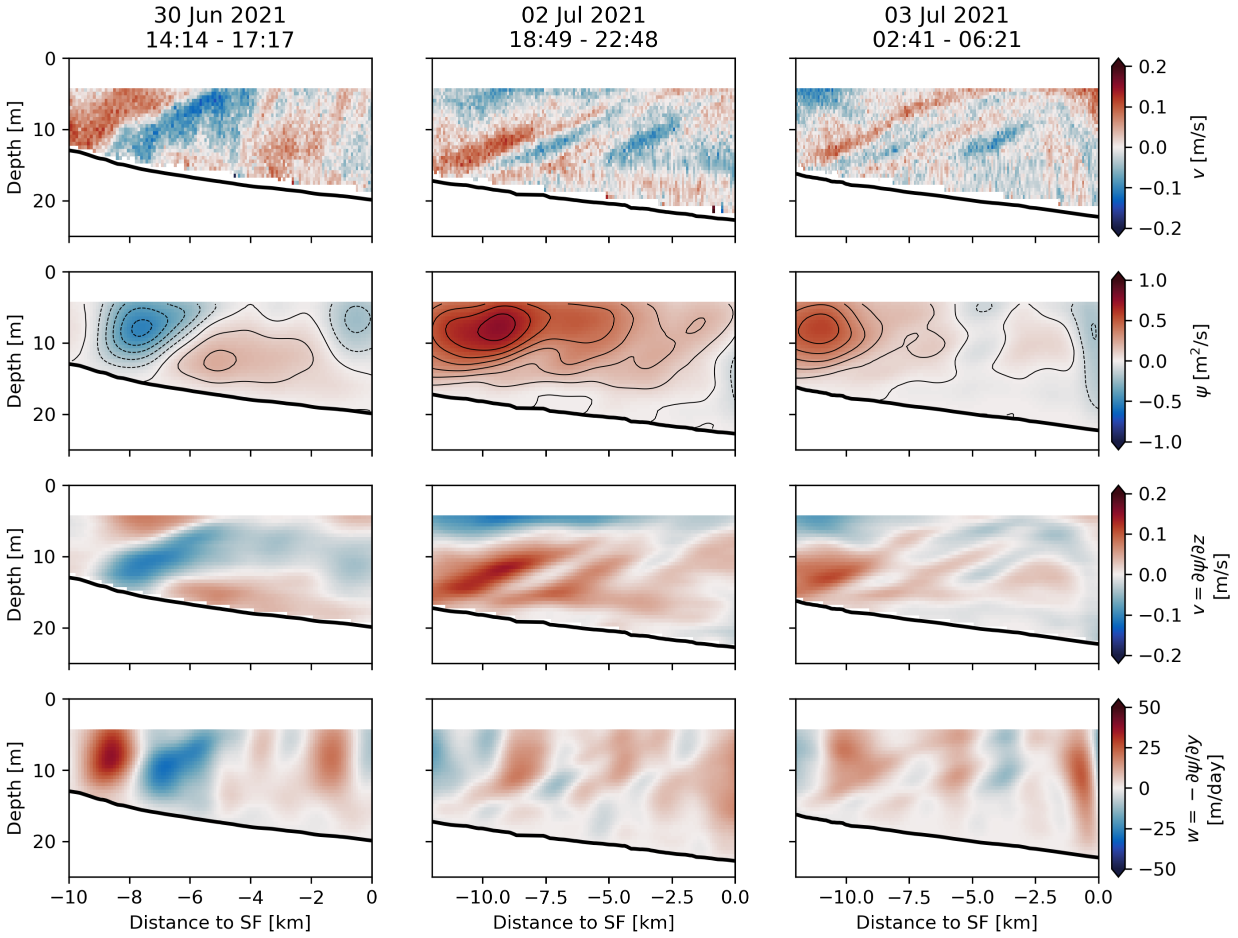


**Figure S8**: **Vertical velocity derived from streamfunction** Rows display (from top to bottom) the observed across-front velocity $v$, the streamfunction $\psi$, the across-front velocity reconstructed from $\psi$, and the vertical velocity derived from $\psi$. Columns correspond to individual cross-front sections; date and time ranges (UTC) are indicated at the top of each column.
